# Evaluation of a sitting light volleyball intervention to adults with physical impairments: qualitative study using social–ecological model

**DOI:** 10.1186/s13102-020-00187-8

**Published:** 2020-07-08

**Authors:** Ka-Man Leung, Pak-Kwong Chung, William Chu

**Affiliations:** 1 Department of Health and Physical Education, Education University of Hong Kong, 10 Lo Ping Rd, Ting Kok, Tai Po, Hong Kong; 2 Department of Sport and Physical Education, Hong Kong Baptist University, Kowloon Tong, Kowloon, Hong Kong

**Keywords:** People with physical impairments, Hong Kong, Intervention, Adaptive, Physical activity, Interviews

## Abstract

**Background:**

This study was a part of 16-week sitting light volleyball (SLVB) intervention program which examined the effects of the intervention on the physical and psychological attributes of adults with physical impairments (PWPI) in Hong Kong. Gaining a deeper understanding of the perceptions and experiences of PWPI in the SLVB intervention is critical to the development of SLVB as a physical activity and a sport. The aims of this study were (a) to assess participants’ experiences of the intervention and (b) to examine the suitability and feasibility of SLVB intervention for PWPI.

**Methods:**

Twenty participants (mean age = 53.52 years, standard deviation 9.02 years; 60% female; 25% with at least a college degree) participated in semi-structured interviews.

**Results:**

Content analysis revealed features of their experiences at the *individual* or *intrapersonal* level (physical and psychological health, enjoyment, novelty, competence, autonomy), *interpersonal* level (socialization and teamwork, social support), *organizational* and *community* levels (perceived sport venue environment, venue accessibility, safety, dissemination of information), and *policy* level (resources allocation by the government). The participants also commented on the suitability and feasibility of the SLVB intervention for PWPI, its content and coaching, the modified rules, the duration of sessions, scheduling, and the number of participants and coaches.

**Conclusions:**

This study identified several themes relevant to the promotion of PWPI engagement with SLVB and demonstrated that adopting a multilevel approach to the intervention resulted in positive outcomes for participants. Playing SLVB is suitable and feasible for PWPI. The findings contribute to the understanding of the experiences PWPI had of the SLVB intervention, which is critical to the further development of SLVB as a physical activity and a sport.

## Background

People with physical impairments (PWPI) tend to be less physically active than those without impairment [1, 2]. In 2015, the Hong Kong Special Administration Region (HKSAR) government commissioned a consultancy study to examine methods to promote PWPI participation in sports. Schools for PWPI expressed concerns regarding the decrease in types of sports and number of courses offered to PWPI [3]. The HKSAR government advocates sports opportunities for all, including PWPI; accordingly, in last years, the HKSAR government has increased the amount of resources allocated to supporting the development of sports for PWPI. Expenditure on the development of sports for PWPI in 2018–2019 was estimated to be HK$70 million, representing an increase of more than 50% compared with the previous year [4].

In addition to promoting existing sports programs, practitioners and researchers [2, 3] should develop new physical activities (PAs) for PWPI. Light volleyball (LVB) is a relatively common PA for older adults in China [5]. Compared with a traditional volleyball, an LVB is larger (with a diameter of 80–83 cm vs. the diameter of 65–67 cm of a traditional volleyball), lighter (100–150 g vs. 250 g), and travels at a lower velocity. Furthermore, an LVB remains airborne for longer than a traditional volleyball does, which increases rally duration between players during games. Leung and colleagues [6] conducted a 15-week LVB intervention to evaluate its effectiveness in improving the health outcomes of adults aged > 60 years in Hong Kong. The participants in the LVB group demonstrated higher cardiovascular endurance, upper extremity muscle strength, and PA enjoyment than participants in the control group. The authors of the pilot study suggested that future studies should investigate the effects of LVB on the health of other populations with relatively low fitness levels (e.g., PWPI).

Sitting volleyball (SVB) is an official Paralympic sport. Similar to a traditional volleyball game, an SVB game comprises two teams of six players. Unlike traditional volleyball, an SVB player’s position is determined by the location of contact between the player’s buttocks and the floor of the court (it is played while sitting on the floor). The British Paralympic Association [7] stated that SVB has a faster pace than an Olympic indoor game because of the modified rules of the game (i.e., lower net height). Thus, SVB is a fast, high-level competitive team sport, demanding power and agility, and is only suitable for PWPI with high sports competence.

With consideration for the high speed and competence requirements of SVB and with the aim of extending the aforementioned work, the objectives of this sitting light volleyball (SLVB) intervention were to develop a new sport to provide additional PA opportunities to PWPI in Hong Kong and improve their health. SLVB modifies LVB and SVB for PWPI. The larger, lighter ball used in SLVB makes it more accessible to PWPI with a low fitness level (i.e., with a slow speed and reaction time). Furthermore, the light weight of the ball results in considerably less soreness in the forearms than that caused by playing with a traditional volleyball. SLVB is a safe, noncontact team sport that can be played on a standard court with an area of 10 m × 6 m or on a space-friendly badminton court. The smaller court enables the promotion of SLVB for PWPI in facilities in Hong Kong that have limited space. Notably, having one nondisabled player is optional. SLVB has modified rules, such as the ball being permitted to bounce once during each pass. Being more inclusive and easier to learn and offering players greater control, SLVB is relatively accessible for individuals with muscular degradation or motor impairment. Leung and colleagues [8] conducted a 16-week SLVB intervention in 2018 and examined its effectiveness in improving health outcomes of PWPI in Hong Kong. The study results revealed that compared with the control group, the SLVB intervention group had significantly higher cardiovascular endurance, stronger body composition, and more reported PA enjoyment. Furthermore, the SLVB intervention effectively enhanced the participants’ quality of life with respect to role limitations due to physical health or emotional problems, body aches, and mental health.

This study was a part of an SLVB intervention. The aims of this study were (a) to assess participants’ experiences of the intervention and (b) to examine the suitability and feasibility of SLVB intervention for PWPI. The findings of the present study contribute to understanding the perceptions and experiences of the participants in SLVB intervention and may help to fine-tune its follow-up studies. This study also offers insights for practitioners who wish to promote SLVB at service centers. All of the aforementioned contributions are critical to the development of SLVB as a PA intervention or program in communities.

## Methods

### Social ecological approach

This study adopted a deductive content analysis, wherein data analysis relied on a social ecological model (SEM) [9]. This model has been used to create more physically active communities in a framework of studies on the sustainability of PWPI empowerment projects in Nairobi, Kenya [10] and to define the social inclusion of people with intellectual and developmental disabilities [11]. The model assists with understanding the multifaceted and interactive effects of personal and environmental factors that influence behaviors and identifying behavioral and organizational leverage points and intermediaries for health promotion within organizations. It has five nested hierarchical levels, namely individual, interpersonal, community, organizational, and policy or enabling environment levels. *Individual factors* refer to personal factors, such as fitness level, that affect an individual’s likelihood of participating in a PA. *Interpersonal factors* refer to the relationships, culture, and society that shape an individual’s interactions (e.g., social support). *Organizational factors* are institutional rules and methods for implementing and promoting a PA, including the organizational capacity (such as the number of PA professionals and therapists) of nongovernmental organizations (NGOs) to organize structured PA programs that are suitable for PWPI. *Community factors* represent PA promotion through collaboration among parties (e.g., NGOs and academic institutions). *Policy factors* include policies regarding the allocation of resources for health promotion and access to health care services and restrictive policies (e.g., high fees for health services) or lack thereof.

### Semi-structured interviews

Data were collected through semi-structured interviews conducted in Cantonese after a 16-week SLVB intervention. The semi-structured format is frequently adopted in qualitative research [12] and health care contexts [13]. These interviews were conducted to collect information on (a) participants’ experiences of the intervention and (b) the suitability and feasibility of the SLVB intervention for PWPI. A set of interview questions was developed by the principal investigator, specifically for the purposes of this study. It was then pilot tested with five PWPI and two investigators (KML and WC) involved in the study (see Additional file 1 for sample questions). The interviewers adapted a flexible approach and, when necessary, altered the question sequence or asked probing questions to facilitate in-depth conversation regarding the intervention. Because semi-structured interviews can combine a predetermined set of open questions with opportunities to further explore particular themes or responses, they were employed to understand the process of the SLVB intervention, its effects, and areas for improvement. Unlike the use of a structured questionnaire, this qualitative method of inquiry does not limit interviewees to a set of predetermined answers and allows them to discuss and raise points that the interviewer may not have considered. This method assisted the interviewees in describing complex phenomena and the interviewers in collecting information on SLVB, immediately clarifying the information collected, and ensuring optimal use of the limited interview time [14].

### Procedure

This study was conducted between September and November 2018. All research activities were reviewed and approved by the University Institutional Review Board (REC/18–19/0378). Additional details and results of the SLVB intervention were provided elsewhere [8]. A cover letter stating details of the study (e.g., aims and procedures) was given to our NGO partner serving PWPI in Hong Kong. During our final visit in the intervention, participants were purposively invited by our research assistant to participate in the present study. Additionally, the manager of the NGO with the right to access the contact information of the SLVB intervention participants published information regarding the study on social media platforms (e.g., WhatsApp) and in the NGO’s bimonthly magazine. Interested participants enrolled in person by contacting the person in charge of the NGO. Details of the interviews (time and venue) were confirmed through discussion between interviewees and interviewers. Before the interview, participants were informed of their confidentiality and details of the study, namely its objectives, their role, potential risks of participation, and their right to leave the project at any time. Participants agreed to participate by signing the consent form. All interviews were conducted by the principal investigator (KML) and a trained research assistant in a meeting room of our partner NGO. The interviewers were female, were knowledgeable of sports and exercise (particularly of LVB), and were trained in qualitative research methods, including interviewing. Field notes were recorded during and after each interview. All interviewees had no prior relationship with the interviewers, except when pretest and posttest measurements were conducted. Other than interviewers and interviewee, no one was presented at the meeting room. All interviews were audiotaped for further data analysis. Participants were provided the opportunity to review the transcript. An HK$100 supermarket voucher was given to participants to acknowledge their contribution to the study. The interviews took an average of 40 min (range = 20–77 min).

### Participants

Twenty-three participants met our inclusion. These were (a) age of ≥18 years, (b) registered as having a physical impairment in the Central Registry for Rehabilitation, (c) presence of at least one functional arm, (d) no diagnosis of cognitive impairment. Because three participants refused to participate for personal reasons, 20 interviews were conducted, and saturation was reached. No repeat interviews were conducted. The interview participants (*n* = 20; 12 women, 25% with a college degree or higher) had a mean age of 53.52 years (standard deviation [SD] = 9.02). They included both completers and noncompleters (the attendance rate in the SLVB intervention was less than 80%). Their attendance in the intervention averaged 66.11%. Participant characteristics are summarized in Table 1.

**Table 1:** Characteristics of Participant

Characteristic	*N*
Sex	
Female	12
Male	8
Age (years)	
< 40	2
40–50	2
51–60	14
61–70	2
Education level	
Primary School Level	1
Secondary School Level	14
University Level and Above	5
Attendance rate	
Completer (attendance rate of intervention was 80% or above; attended 26 training sessions or above)	10
Non-completer (attendance rate of intervention was 79% or less; attend less than 26 training sessions)	10

*Note*. *N* number of participants

### Data analysis

All interviews were transcribed verbatim and checked against the coinvestigators’ notes. The transcripts were qualitatively analyzed using thematic analysis. This type of analysis is one of a cluster of analytic approaches to identifying patterns of meaning across a qualitative data set. Both theory-driven and deductive thematic analysis were used to analyze data and consolidate them into meaningful themes, following the process suggested by Biddle et al. [15]. A coding team comprising two individuals (KML and CYM) read all transcripts and developed a codebook, which was iteratively refined throughout the coding process and with reference to the Social Ecological Model. Each transcript was coded independently by two coders using the most recent codebook. At regular intervals throughout the process, a reliability review of the coding was conducted by the team. Any differences were resolved through discussion to reach a consensus. The final codes were determined by KML, who ensured that the transcripts coded earlier reconciled with the most recent codebook.

The raw data extracted for thematic analysis included participant quotations and our corresponding interpretations. Measures used to improve the trustworthiness of the analysis included inviting both completers and noncompleters to participate in the study and asking participants to interpret the data so as to increase the credibility of the results. The 32-point consolidated criteria for reporting qualitative studies were used to report the results [16].

## Results

### Participants’ experiences of the intervention

#### At the individual or intrapersonal level

##### Physical and psychological health

Reduction of lumbar spine pain and improvements in body coordination were the positive outcomes perceived by the participants.

One of the male participants (M4) stated that SLVB helped to reduce lumbar spine pain: *“I often sat on the floor while playing SLVB. Eventually, I found that my lumbar spine and hip bones had become more flexible than before. I used to have lumbar spine pain. After playing SLVB, I found it less painful than before.”*

Two male participants, (M6) and (M2), stated that SLVB was good for physical coordination: *“We have to rotate and manage hand–eye coordination … Hand–eye coordination is important for catching a ball”* and *“It demands reactivity [reaction] and synchronization of the players … As such, I think it is good for the growth of the brain and body.”*

In addition to physical health, potential improvements in psychological health (positive mood and reduce anxiety) and quality of sleep were mentioned by some participants.

A female participant (F5) reported *“Playing SLVB puts me in a good mood. I become worry-free when I’m focused on the ball.”*

##### Enjoyment

Analysis of the semi-structured interviews indicated participants’ perceived enjoyment when playing SLVB.

One male participant (M3) stated *“The sport ground was full of happiness and laughter. People cheered when someone had a good serve. They laughed when someone hit the ball out of bounds. The atmosphere was joyous. (How about you?) I felt happy too.”*

##### Novelty

Because SLVB was new to the participants, they were curious and eager to learn what it was, how it was played, and whether they could participate in this form of PA.

One female participant (F1) stated *“SLVB was new to me. The moment I first heard of SLVB, I wondered if it can be played by wheelchair users like me. I was so curious to find out how it is played. Moving around the volleyball court, hitting a ball on my wheelchair, and so on are totally new to me. All this attracted me to the project.”*

One of the male participants (M1) said *“I wanted to know more about SLVB as a sport … I learned how to play a new kind of sport.”*

##### Competence

The participants indicated that playing SLVB promoted a sense of competence derived not only from successful service, scoring points, and progressively mastering techniques but also from positive feedback from coaches.

One female participant (F4) said *“I felt very happy when I served or received a ball well.”* Another male participant (M4) stated, *“If I hit a good ball, Sir Y.W. would praise*.”

##### Autonomy

The participants stated that they had attained a gain in bodily autonomy and a sense of freedom from playing SLVB.

Bodily autonomy and sense of freedom were felt by a male participant (M6). He reflected: *“After the caliper is removed, I can play it, rotate, and pick up the ball freely … Now, watching the videos, I found myself so free … I don’t need a wheelchair or caliper, which is so delightful. I also feel like I’m in a dream as if I am an astronaut, no limitations and no caliper.”*

#### At the interpersonal level

##### Socialization and teamwork

In addition to making references to their physical and psychological health, the interviewees also described social interaction effects from participating in the SLVB intervention. Elements of teamwork such as mutual inclusion, cooperation, team spirit, and combined action as a group were described.

A male participant (M1) mentioned “*The good things are ... interacting with my friends. With my teammates, we discussed how to win a game strategically and adapt ourselves to play better.”*

A female participant (F2) said *“Individual exercise differs from group exercise … Group exercise demands mutual inclusion, cooperation, and interaction among participants. For SLVB, you need at least 10 people to make it fun.”*

##### Social support

Support from peers, friends, family members, coaches, and volunteers was evident during the intervention.

Support from peers, friends, and family was described by a female participant (F2) as follows: *“Some of my classmates are very friendly. They are proactive and say “hello” to me and even invite me to play with them in a small group. They told me about some tricks for playing SLVB, which helped me do better.”* A male participant (M2) stated *“Most of them encouraged me to do so. My wife supported me too, even though she is more introverted than me.”*

Support and positive feedback from coaches and volunteers were evident to a male participant (M4): *“Sir Y.W. would praise me and say, ‘Well done, you have applied the skills I taught you.’ I got so much satisfaction from this positive feedback.”* A female participant (F4) stated *“I am very thankful to the ball boys and girls ... They helped by picking up the balls for us quickly. They did a great job.”*

#### At organizational and community levels

##### Perceived sports venue environment

Venue comfort, privacy, spaciousness, and convenience positively affected engagement in the intervention.

One male participant (M4) exclaimed “*Superb, superb! Unlike other play areas, there were no other players except us. I liked the privacy and comfort this facility gave me very much.”*

Accommodating numerous wheelchairs and participants’ belongings required sufficient space. One male participant (M6) commented on the venue: *“The center is convenient and comfortable inside. It is big enough to store our wheelchairs and the air conditioning is great.”*

##### Venue accessibility

Because many participants were wheelchair users and had walking impairments, accessibility concerns were a central part of discussions. The accessibility of a venue is vital to encouraging participation in a PA. One male participant (M3) said *“Many of them had become proactive in playing SLVB, even though the venue was quite inconvenient for them to get to, given the steep road, insufficient parking spaces, and so on.”*

##### Safety

Safety is a concern when playing sports, particularly for PWPI and people with relatively low fitness levels. When questioned regarding the potential risks of playing SLVB, a male participant (M3) stated “*Every sport has its risks. SLVB players may strain their muscles. They may fall to the floor and get hurt if they don’t play it properly. At their ages, the chance of them hurting themselves is higher. It would be troublesome if they hurt their hands.”*

Nevertheless, risk can be reduced by using proper techniques and protective gear, as mentioned by another male participant (M3): *“Some people have a weak lumbar region. They will fall straight down onto the floor when they lose their balance. As you said before, they should have been taught how to fall properly so as not to hurt themselves, such as how to use elbow supports as buffers while falling.”*

##### Dissemination of information

One female participant (F2) commented on the dissemination of information: *“The enrolment process was smooth. However, I sometimes found that the dissemination of information was quite confusing ... It would be better if we could be informed as a group by a single party who has all our phone numbers.”*

#### At the policy level

##### Resources allocated by the government

Concerns regarding the sustainability of activity sessions were raised by participants, who wanted to see local government departments provide resources to support activity sessions such as by providing community facilities to play SLVB. The lack of wheelchair-accessible facilities in the community discouraged participation in SLVB. One female participant (F1) stated *“I rarely go out … for extra exercise … some places have staircases and are therefore not suitable for a wheelchair.”*

**Fig. 1:**
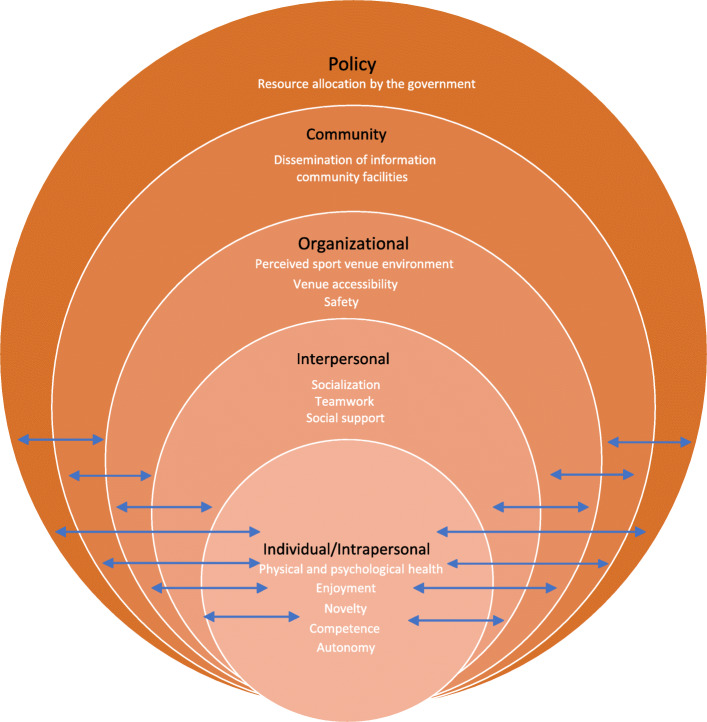
Social ecological model developed for the study

She further commented *“Many sports centers are booked for basketball and badminton, leaving no room for us to play SLVB. Alternatively, I think the government should let us book community halls to play SLVB. Community halls are big.”* Supporting quotations are presented in Table 2. The model developed using the findings is presented in Fig. 1.

**Table 2:** Supporting Quotes about Participants’ Experiences

SEM Levels and themes	Participant quote	Participants
**Individual/intrapersonal level**		
*Physical and psychological health*	*“It enhanced my body strength as well. When the receiving team wins every rally, it gains the right to serve and rotates before actually serving … This rotation movement is even more tiresome than serving and smashing a ball. It strained my arms more than wheel-chairing did. This type of lateral movement, through the use of different muscles, strengthened my arms over time. “*	M4
	*“We are limited by our disabilities to do many things such as running. SLVB gives us an opportunity to do aerobic exercise. It is also good for improving our reaction time.”*	F2
	*“I feel more energetic. Besides, I slept well after playing SLVB. Yes. I think it has improved the quality of my sleep.”*	F3
*Enjoyment*	*“I would tell them that SLVB is something I never came across before. Playing SLVB is very joyful and sort of doing exercise. I seldom did exercise before I retired.”*	F2
	*“In general, I found SLVB fun-full to play with.”*	F4
*Novelty*	*“It was new to me. I never did it before.”*	F2
	*“I have done other exercises for at least 3 years. I never did SLVB. That is why I want to try.”*	F2
	*“I want to know what SLVB is about. I want to do more exercise for a healthy and strong body.”*	M3
*Competence*	*“As a matter of fact, I am a fan of VB. Have you ever heard of Lang Ping? I like to watch her playing VB. I know the rules of the games. However, I have never played VB because of my disabilities and body weight. Thanks to the project, I am able to play SLVB.”*	F1
	*“I feel happier because I can make it. I can play!”*	F4
*Autonomy*	*“Suitable. For instance, some players can do SLVB with just one hand. Despite our walking impairments, we are able to play SLVB in a sitting position. I do not know how to operate and control a wheelchair. I fear of falling if playing SLVB on a wheelchair. Playing in a sitting position fits me very much.”*	F2
**Interpersonal level**		
*Socialization and teamwork*	“*I felt quite happy to play and practice passing the ball in a small group … ... I am happy to know a few new friends too.”*	F2
	*“Absolutely, we played hard to win as a team. We cheered to each other whenever scoring a point. We were very happy as a team. It is awesome.”*	F1
	*“To me, SLVB is playful. It cultivates team spirit and demands cooperation of each member …*.”	M3
	*“SLVB provides us with a chance to move around, stretch our muscles, enable communication within the team, learn how to interact with each other, and to follow instructions from the coaches. There are many advantages …*.”	M3
*Social support*	*“I didn’t tell others except for my old friends. They said that wow, you were awesome!”.*	F2
	“*Yes, they (family members) were very supportive. They said that it was good for me to go out and play more. They told me not to stay at home alone.”*	F2
	“*I felt that I have become more proactive than before (in knowing friends and social interactions).”*	F2
	*“I didn’t hear any objection. Both my family and daughter were supportive.”*	F3
**Organizational / Community levels**		
*Perceived sport venue environment*	*“The playground is big and the indoor environment is ok.”*	F1
*Venue accessibility*	*“It is quite convenient to come here by train (Kowloon MTR station) and bus. Furthermore, there is a public car park at the entrance. However, the steep roads leading to the sportsground are not convenient for wheelchair users.”*	M3
	*“Given my mobility, I can manage to off-load or up-load my wheelchair even though the parking lot is a bit narrow. No big deal.”*	M2
*Safety*	*“As I have walking impairment on one leg only, one side of my buttock is bigger than the other. As a result, I need to balance myself with one hand while sitting on the floor. This explained why I seldom use two but mostly one hand to receive the ball. Otherwise, I would lose my balance and fall backward. Besides, I could not bend my legs while sitting on the floor. That’s why my legs would cramp sometimes.”*	F2
	*“SLVB is not that risky. The risk of falling down could be higher if we played while standing up. While sitting on the floor, the risk of falling and hurting our hands is lower. The risk could be lowered by wearing elbow-supporters. The only potential risk is that our legs are powerless. Some team members suffered from abrasion while dragging or moving their legs on the floor.”*	F1
*Dissemination of information*	*“Some players may disagree with setting up a WhatsApp group because they don’t like to receive irrelevant personal messages. However, I think that the Group should only be used by the responsible party to disseminate relevant information such as change in venue, training schedule,* etc. *One message will then be sent to all.”*	F2
**Policy Level**		
*Resources allocation by the government*	*“Unlike wheelchair rugby and basketball, SLVB is played without a tailor-made wheelchair and in a sitting position. As such, I think it is ok and worthy of promoting SLVB as long as a sportsground is available.”*	M3

#### Suitability and feasibility of SLVB intervention elements

##### Suitability

SLVB is played in the sitting position and involves movements with relatively low strain and intensity; hence, it was perceived to be suitable for PWPI.

A female participant (F3) stated *“As you know, most HKFHY members are disabled in their legs. Exercises requiring a lot of leg movements don’t suit us. Because it is played in a sitting position, SLVB is suitable for us.”*

##### Project content and coaching

In general, the project feasibility was perceived to be adequate, time-appropriate, and progressive, and the caring approach adopted by the coaches was appropriate and made instructions easy to understand.

One male participant (M2) said that the content of the project *“was adequate. It takes time for you to progress from not knowing to knowing how to play ... The duration and progression of the project were good.”* He (M2) added *“I think that the two coaches started with the basics and then gradually taught us more techniques for playing SLVB (step by step approach). It should be OK.”* Participant (M6) reflected that *“It has a theoretical basis and is taught in a structured way. It is easily understood.”*

## Discussion

This qualitative study aimed to (a) assess participants’ experiences of the intervention and (b) assess the suitability and feasibility of SLVB intervention for PWPI. A Social Ecological Model was used in this qualitative study and yielded five levels of themes that influenced PWPI to engage in the SLVB intervention.

### Participants’ experiences of participating in the intervention

#### At the individual or intrapersonal level

##### Physical and psychological health

The most frequently cited outcomes of the intervention were participants’ perceived improvements in their physical health, such as in hand–eye coordination. These aligned with the results of the meta-synthesis, wherein health was a substantial concept. The potential for improvement in health and well-being motivated engagement with the intervention, and these improvements facilitated the long-term maintenance of a physically active lifestyle [17].

##### Enjoyment and novelty

Feedback from the participants of the present study indicated that the promotion of SLVB in the form of group participation in a PA promoted the subjective happiness of PWPI. Participants expressed an overall perception that they felt happy and joyous (individually and as a group) when playing SLVB, which is consistent with the findings of a previous study wherein “enjoyment” was mentioned most often in relation to PAs and was evident in both group and individual activities [17]. The uniqueness of SLVB was noteworthy and introduced novelty (an outcome) to the participants. This is consistent with the reports of previous studies [2, 17] that diverse, adapted, or new PAs are urgently required for PWPI in the community.

##### Competence and autonomy

Competence and autonomy are concepts derived from self-determination theory [18]. Many of the participants expressed that they derived a sense of competence from making a good serve, gradually mastering SLVB skills, receiving positive feedback from coaches, and even helping peers progress. Many also reported they gained bodily autonomy and a sense of freedom by playing SLVB, suggesting that they overcame barriers to exercise through independent action. Among PWPI, the dependency of their mobility on external factors causes feelings of a lack of control over their body, self, or life [18, 19].

#### At the interpersonal level

Consistent with research on PWPI [20] and experts who work for people with disabilities [21] that has addressed the requirements of a PA intervention for PWPI, the present study highlights the need for social interaction during PAs. People participating in sports interact with each other through teamwork, thereby developing social skills and strengthening their social development [22, 23]. The results of the present study indicate that social interaction effects (socialization, teamwork, and social support) contributed to the effectiveness of the SLVB intervention.

#### At the organizational /community levels

The environment in which a PA takes place influences participant engagement in an intervention. For example, an unsupportive environment and inaccessible facilities and transport were reported to inhibit continued exercise [24]. Our findings echo those of the aforementioned study in that a positively perceived sports venue environment (with regard to comfort, privacy, spaciousness, and accessibility) was necessary for engaging PWPI in our intervention.

##### Safety

Safety is certainly a concern when playing sports, particularly for PWPI and people with a relatively low fitness level. Our findings reveal that participants were vulnerable to loss of balance, falling, and age-related injury (the mean participant age was 53.32 years). Nevertheless, the use of proper techniques (e.g., how to prevent falling and how to fall safely) and protective gear (e.g., elbow, ankle, and knee supports to protect against abrasion) can reduce risks.

##### Dissemination of information

Given the importance of communication, participants should be enabled to communicate with each other through a variety of means that meet individual preferences and capabilities [17]. One participant opined that a specific WhatsApp group should have been used by the responsible party to disseminate relevant information such as changes of venue or the training schedule.

### Community facilities

#### At the policy level

Resource allocation such as through the provision of sports facilities was identified by NGOs as one of the obstacles to promoting sports participation in the community. Inaccessible facilities hinder continued exercise [17]. Our findings also revealed that a lack of wheelchair-friendly and accessible facilities in the community (whether public sports complexes or NGOs) discouraged future participation in SLVB.

### Suitability and feasibility of the content of the SLVB intervention

The interview results supported the expected outcomes that SLVB was considered by many participants (both female and male) to be suitable for PWPI mainly because it is played in a sitting position and is less vigorous and intensive than wheelchair basketball; a player’s hands are less likely to be injured. Their comments regarding the rules and content of SVLB reflected that our modifications to SVB and LVB seemed reasonable to them. Participants expressed satisfaction with the content, coaching, scheduling, and class size of the intervention.

## Conclusion

In this qualitative study, the experiences of PWPI who participated in a 16-week SLVB intervention and the suitability and feasibility of the intervention for PWPI were examined. A SEM was used, yielding five levels (individual, interpersonal, organizational, community, and policy) of themes that influenced PWPI engagement in the intervention. The model indicated that these theme levels are interrelated. For instance, enjoyment at the individual level might derive from socialization at the organizational level. In general, the model demonstrated that adopting a multilevel approach to the intervention resulted in positive outcomes for the participants.

Regarding this study’s strengths, participants who completed and those who did not complete the intervention program were interviewed, which enhanced the rigour of the conclusions. Furthermore, a theory-driven framework guided the study. However, the study had limitations. First, exit rather than interim interviews were conducted, which inhibited interim improvements of the intervention program. Second, all participants belonged to one partner NGO, which might have encouraged groupthink.

Our findings provide practical insights into issues central to mapping out future or specifically adapted PA (e.g., SLVB) intervention programs for PWPI. For example, researchers and practitioners may consider the inclusion of protective gear to reduce the risk of injury and the provision of a specific communication channel (e.g., a WhatsApp group) for dissemination of information among the participants. In addition, areas for future exploration were identified, such as whether improved engagement in SLVB can be realized by recruiting participants from multiple NGOs, recruiting more participants, and ensuring an equal sex ratio.

In sum, the findings contribute to understanding of the perceptions and experiences of PWPI in the SLVB intervention, which is critical to the development of SLVB as a community PA intervention or program.

## Supplementary information

**Additional file 1.** : Appendix 1. interview guide

## Data Availability

The datasets generated and analyzed during the current study are not publicly available due to ethical restrictions but are available from the corresponding author on reasonable request.

## Abbreviations

SLVB: Sitting light volleyball
SEM: Social ecological model
PWPI: People with physical impairments
PAs: Physical activities
HKSAR: Hong Kong Special Administration Region
LVB: Light volleyball
SVB: Sitting volleyball
NGO: Nongovernmental organization
