# Supplementary material for: Evaluation of a sitting light volleyball intervention to adults with physical impairments: qualitative study using social–ecological model
Source: BMC Sports Sci Med Rehabil. 2020 Jul 8;12:41. doi: 10.1186/s13102-020-00187-8 (PMC7346623; doi:10.1186/s13102-020-00187-8)
Supplement: Supplementary file 1 — Additional file 1. : Appendix 1. interview guide [file 13102_2020_187_MOESM1_ESM.docx]

Appendix 1. Sample Interview Guide

**Individual level**

1. Why did you enroll our LVB program? What was (were) your main motivator(s) to participate in our SLVB intervention program in the program?
2. What did you think about your competence on playing SLVB?
3. What did you think about the health benefits/risk playing SLVB?
4. Did you feel motivated to play SLVB during the intervention program?
5. What did you enjoy most about playing SLVB?
6. What did you like least about playing SLVB? – Did you encounter any difficulties?
7. Was(were) there any obstacle(s) preventing you from attending the SLVB class?
8. Were there any changes in your physical activity’s engagement after the intervention program?

**Interpersonal levels**

1. What did you think about the social benefits/concerns playing SLVB?
2. What would you think your significant relatives and friends think of you participating in the SLVB program or any other physical activities?
3. Did any one of them specifically encourage or discourage you to play SLVB? Who is he/she?
4. How did they encourage/discourage you? (Probe: emotional/physical/instrumental support…)

**Other levels**

1. Are you satisfied with the physical environment of where you played SLVB?
2. Was there anything about the physical environment of the program that you think stop/promote you from playing SLVB?
3. What sort of environment of this program encouraged you to play SLVB??
4. What sort of environment of this program discouraged you to play SLVB??
5. How was (were) the mentioned environment(s) stop/promote you from playing SLVB?
6. Did you think the administrative processes are well-organized? (e.g. Recruitment? Enrollment? Membership? Others?)
7. Were there any administrative issues that encourage/discourage you to play SLVB?
8. What aspect of your culture/social beliefs in your community would influence you to play SLVB?

**Suitability and feasibility of the SLVB program**

1. Please share with me your experiences and thoughts about the SLVB program?
2. How did you think about the SLVB program, especially for people who are physically impairments?
3. Was the content of program sufficient? Inspiring? Difficult?
4. How was the design of the SLVB program? (For example, Length? Time? Venue? Class size...etc.)
5. How was the performance of our SLVB coaches?
6. What could be improved upon? Was there anything missing that you think the program should include? (e.g., special SLVB content or other related content)
